# Use of Cancer Screening Tests, United States, 2023

**DOI:** 10.5888/pcd22.250139

**Published:** 2025-08-14

**Authors:** Susan A. Sabatino, Trevor D. Thompson, Jennifer M. Croswell, Maria A. Villarroel, Juan L. Rodriguez, Emily E. Adam, Lisa C. Richardson

**Affiliations:** 1 Division of Cancer Prevention and Control, National Center for Chronic Disease Prevention and Health Promotion, Centers for Disease Control and Prevention, Atlanta, Georgia; 2 Division of Cancer Control and Population Sciences, National Cancer Institute, Bethesda, Maryland; 3 Division of Health Interview Statistics, National Center for Health Statistics, Centers for Disease Control and Prevention, Hyattsville, Maryland; 4 Division of Population Health, National Center for Chronic Disease Prevention and Health Promotion, Centers for Disease Control and Prevention, Atlanta, Georgia

## Abstract

**Introduction:**

The objective of this analysis was to provide national estimates for use of breast, cervical, and colorectal cancer (CRC) screening tests, including for the recently expanded CRC screening age group (ages 45–75 y).

**Methods:**

We used data from the 2023 National Health Interview Survey to estimate proportions of screening-eligible adults up to date with breast (women aged 50–74 y), cervical (women aged 21–65 y), and CRC screening (adults aged 45–75 y). We compared breast and CRC estimates age-standardized to the 2000 US standard population to Healthy People 2030 (HP2030) targets. Age-standardized estimates of breast, cervical, and CRC test use were compared with estimates from 2019 (breast, cervical) and 2021 (breast, cervical, CRC).

**Results:**

In 2023, estimated percentages of adults up to date were 80.0% (95% CI, 78.7%–81.2%), 75.4% (95% CI, 74.1%–76.6%), and 67.4% (95% CI, 66.3%–68.4%), for breast, cervical, and CRC screening test use, respectively. CRC test use was lower among those aged 45 to 49 years than those aged 50 to 75 years (37.1% vs 73.4%, *P* < .001). Mammography use approximated the HP2030 target. CRC test use was below the target. Breast, cervical, and CRC screening test use varied with almost all sociodemographic characteristics and health care access, financial hardship, and other barriers examined. Mammography estimates were somewhat higher and cervical test estimates were lower in 2023 than in 2019 and 2021. CRC test use was lower in 2023 than 2021.

**Conclusion:**

In 2023, most adults were up to date with breast, cervical, and CRC screening test use; however, 1 in 3 adults (CRC) to 1 in 5 adults (breast) were not. Future monitoring can help determine if changes continue and track progress toward national targets.

**Summary**

**What is already known on this topic?**

Breast, cervical, and colorectal (CRC) screening is recommended. Use is below national targets and declined during the COVID-19 pandemic.

**What is added by this report?**

In 2023, most adults were up to date with breast, cervical, and CRC screening test use, although 1 in 3 adults (CRC) to 1 in 5 adults (breast) were not. Mammography increased and cervical test use decreased in 2023. CRC screening test use among those aged 45 to 49 years in 2023 was low. People with less access to health care, financial hardship, and other barriers generally also had lower use.

**What are the implications for public health practice?**

Future monitoring can help determine if changes continue and may inform evidence-based interventions to increase use.

## Introduction

Breast, cervical, and colorectal cancers (CRCs) accounted for more than 426,000 cancer diagnoses and almost 100,000 deaths in 2021 (1). The US Preventive Services Task Force (USPSTF) recommends screening for these cancers to reduce cancer mortality (2), and Healthy People 2030 (HP2030) sets national screening targets (3). In 2021, use of these screenings was below HP2030 targets (4), albeit early in the decade for these targets. Screening use in 2021 might reflect declines during the COVID-19 pandemic (5,6), which have raised concerns about potential effects on cancer outcomes (5–7). Fecal occult blood test (FOBT) or fecal immunochemical test (FIT) use may have increased during that time possibly due to changes in home-based screening test use (4,8), although at least 1 study reported otherwise (9). Although primary care visits declined during the pandemic, some health plans and others, such as health centers, may have increased efforts to make stool-based tests or educational materials available to patients in other ways, such as by mail or online (10). How breast, cervical, and CRC screening test use may have changed after 2021–2022 is less certain.

Several screening recommendations and targets have been revised since 2021. In May 2021, USPSTF expanded recommended CRC screening ages from 50 to 75 years to 45 to 75 years (2). USPSTF also expanded its breast cancer screening recommendations in 2024 to include ages 40 to 49 years (2). In 2023, Healthy People revised 2030 targets for breast (from 80.5% to 80.3%), cervical (from 84.3% to 79.2%), and CRC screening (from 74.4% to 68.3%) (3). The National Health Interview Survey (NHIS) is used to track progress toward these targets (3). This descriptive report updates previous NHIS cancer screening reports (4,11–15) to 1) provide the most recent national estimates of screening test use, including for the first time estimates for the new CRC screening age group and an initial estimate for breast cancer screening among women in their forties, 2) examine differences in use, 3) compare estimates with revised HP2030 targets, and 4) examine use over time.

## Methods

We analyzed data from the 2023 NHIS, the most recent year providing information about breast, cervical, and CRC screening test use. NHIS is an in-person cross-sectional household survey of a nationally representative sample of the civilian noninstitutionalized US population (www.cdc.gov/nchs/nhis). From each household, 1 adult is randomly sampled to provide detailed health information. The final sample adult response rate in 2023 was 47% (16).

Breast cancer screening analyses included women aged 50 to 74 years (n = 6,829); we separately examined use for ages 40 to 49 years as a preliminary estimate before the expanded 2024 USPSTF recommendation. Cervical cancer screening analyses included women aged 21 to 65 years (n = 10,475). CRC screening analyses included respondents aged 45 to 75 years (n = 15,092). Exclusions included personal history of the cancer being screened for or missing cancer history (n = 417 for breast, n = 135 for cervical, n = 147 for CRC); unknown screening status (n = 130 for breast, n = 279 for cervical, n = 331 for CRC); and for cervical screening, prior or unknown hysterectomy (n = 1,334).

Screening test questions asked whether respondents had ever received the test and time since their most recent test. We defined up to date as having received tests within recommended screening intervals (2), including mammography within 2 years (breast cancer screening) and colonoscopy within 10 years, FOBT/FIT within 1 year, computed tomography (CT) colonography or flexible sigmoidoscopy within 5 years, or FIT-DNA within 3 years (all for CRC screening). Cervical screening questions in 2023 included “There are two different kinds of tests to check for cervical cancer. One is a Pap smear or Pap test, and the other is the HPV or human papillomavirus test. Have you ever had a test or tests to check for cervical cancer?” and “When did you have your most recent test to check for cervical cancer?” Up to date was defined as having received a test within 3 years for ages 21 to 29 years, or within 5 years for ages 30 to 65 years. USPSTF recommends Pap testing every 3 years for ages 21 to 29 years and Pap testing every 3 years or HPV alone or with a Pap test (co-testing) every 5 years for ages 30 to 65 years (2).

We examined screening test use by age, sex (CRC screening), race, ethnicity, education, income (percentage of federal poverty threshold), duration of US residence, county metropolitan status, health insurance coverage and type, disability status, having a usual source of health care, difficulty doing errands alone, or a wellness visit within 3 years. We used NHIS imputed income files that include missing income data imputed by multiple imputation (16). NHIS county metropolitan status is based on the 2013 National Center for Health Statistics (NCHS) Urban-Rural Classification Scheme for Counties and categorized into 4 groups (16). We stratified health insurance coverage by age (<65 vs ≥65 y) because of differences in coverage eligibility. Respondents with only Indian Health Service coverage or single service coverage are considered uninsured in NHIS (16). The NHIS disability status composite indicator classifies disability as “a lot of difficulty” or inability to do at least 1 of 6 domains from the Washington Group on Disability Statistics Short Set on Functioning (16). Similar to previously proposed measures and analyses (17,18), other questions included whether respondents had recently experienced food insecurity, worried about paying medical bills should accident or illness occur, and whether in the prior year they lacked reliable transportation, were unable to pay housing and utility costs, had problems paying medical bills, and delayed or did not receive needed medical care because of cost. The NHIS food security indicator is based on responses to 10 questions regarding the prior 30 days (16).

We present unadjusted estimates and estimates age-standardized to the 2000 US standard population. We used age-standardized estimates to compare with HP2030 targets. We did not compare cervical screening test use with the HP2030 target because of differences in defining the calculations for up to date (3). For use over time, we calculated age-standardized estimates of being up to date for 2019, 2021, and 2023 based on USPSTF recommendations in effect for each year. Because USPSTF updated recommended CRC screening ages in mid-2021, we included adults aged 50 to 75 years for 2021. We did not present a CRC estimate for 2019 because information about most recent FIT/DNA was not released (19). Questions about FOBT/FIT use in 2019 were asked only of those aged 40 years or older reporting CRC screening tests other than colonoscopy or sigmoidoscopy. The 2023 NHIS did not ask about type of test (Pap or HPV) received for cervical screening; therefore, we defined up to date for each year as described for 2023. Refinements to nonresponse adjustments and calibration methods for survey weights have occurred over time (16). Differences over time in interviews conducted at least partially by telephone were also present, with these interviews less common in 2019 than in subsequent years (16).

We used Wald *F* tests to test for differences in estimates for 2023 within recommended screening age groups. Survey weights and design variables were used in all analyses. We suppressed estimates not meeting NCHS reliability standards (20). We used the *surveytable* package in R version 4.4.0 (R Foundation for Statistical Computing) and SUDAAN version 11.0.1 (RTI International) to conduct analyses.

## Results

In 2023, 80.0% of women aged 50 to 74 years were up to date with breast cancer screening (age-standardized, 79.8%) (Table 1). Among women aged 40 to 49 years, 62.1% had received a mammogram within 2 years (age-standardized, 61.9%). For cervical cancer screening, 75.4% were up to date (age-standardized, 75.8%), and for CRC screening, 67.4% were up to date (age-standardized, 63.5%) (Tables 2). For CRC screening, those aged 45 to 49 years were less likely than those aged 50 to 75 years to be up to date (37.1% vs 73.4%). Breast cancer screening test use approximated the HP2030 target of 80.3%, although CRC screening test use was below the target of 68.3%.

**Table 1:** Percentage of Screening-Eligible Adults Who Were Up to Date With US Preventive Services Task Force Breast and Cervical Cancer Screening Recommendations, United States, 2023^a^^,^ b

Characteristic	Breast cancer screening					Cervical cancer screening, aged 21–65 y		
	Aged 40–49 y^c^		Aged 50–74 y					
	No.	% (95% CI)	No.	% (95% CI)	*P* d	No.	% (95% CI)	*P* d
Overall (crude)	2,091	62.1 (59.6–64.6)	6,282	80.0 (78.7–81.2)	NA	8,727	75.4 (74.1–76.6)	NA
Overall (age standardized^e^)	2,091	61.9 (59.4–64.3)	6,282	79.8 (78.5–81.1)	NA	8,727	75.8 (74.6–77.0)	NA
Age, y								
21–29	NA	NA	NA	NA	.04	1,565	63.7 (60.8–66.5)	<.001
30–39	NA	NA	NA	NA		2,418	82.7 (80.8–84.6)	
40–49	2,091	62.1 (59.6–64.6)	NA	NA		1,836	76.5 (73.9–78.9)	
50–65	NA	NA	NA	NA		2,908	77.6 (75.9–79.3)	
50–64	NA	NA	3,562	79.0 (77.3–80.6)		NA	NA	
65–74	NA	NA	2,720	81.6 (79.7–83.4)		NA	NA	
Race								
AIAN^f^	—g	—g	118	75.9 (66.5–83.8)	.001	192	77.9 (69.1–85.2)	.001
Asian	166	68.1 (59.1–76.3)	259	80.3 (73.6–85.9)		640	67.8 (62.8–72.5)	
Black/African American	271	71.1 (64.6–77.1)	802	85.6 (82.4–88.5)		1,080	72.7 (69.1–76.2)	
White	1,392	61.2 (58.0–64.4)	4,806	79.0 (77.6–80.4)		6,039	77.5 (76.1–78.8)	
Other single/multiple race	—g	—g	53	73.2 (56.4–86.2)		162	78.1 (69.1–85.5)	
Missing/Unknown	177	54.1 (46.0–62.0)	244	81.5 (75.5–86.6)		614	67.5 (62.8–72.0)	
Ethnicity^h^								
Non-Hispanic	1,671	64.1 (61.5–66.7)	5,567	80.3 (79.0–81.6)	.18	7,054	77.5 (76.1–78.9)	<.001
Hispanic	420	54.6 (48.8–60.4)	715	77.6 (73.6–81.3)		1,673	67.1 (64.2–69.9)	
Mexican/Mexican American	236	52.5 (44.8–60.1)	375	77.2 (71.5–82.3)		952	66.0 (62.0–69.9)	
Other Hispanic	176	58.2 (48.7–67.2)	331	77.8 (71.6–83.2)		702	69.4 (65.2–73.4)	
Unknown	—g	—g	—g	—g		—g	—g	
Education								
Less than high school	157	54.3 (44.6–63.9)	543	70.4 (65.8–74.7)	<.001	585	62.3 (57.4–67.0)	<.001
High school/GED	394	53.4 (47.6–59.1)	1,493	75.7 (73.0–78.3)		1,788	67.2 (64.5–69.8)	
Some college	519	59.7 (54.6–64.6)	1,911	80.3 (78.3–82.2)		2,341	74.7 (72.3–77.0)	
College degree	1,013	70.6 (67.4–73.7)	2,303	86.1 (84.5–87.6)		3,971	83.5 (81.9–84.9)	
Missing/Unknown	—g	—g	—g	—g		—g	—g	
% Federal poverty threshold								
≤138	379	48.4 (42.1–54.7)	1,119	69.0 (65.5–72.4)	<.001	1681	65.5 (62.4–68.5)	<.001
>138–250	384	56.6 (50.4–62.6)	1,234	75.8 (72.6–78.9)		1,577	69.1 (66.2–71.9)	
>250–400	420	62.3 (56.4–68.0)	1,321	81.3 (78.5–83.9)		1,800	74.6 (71.8–77.3)	
>400	908	70.4 (66.8–73.8)	2609	85.2 (83.6–86.7)		3,669	83.2 (81.5–84.9)	
Duration of US residence								
<10 y	104	51.6 (40.5–62.7)	62	68.0 (52.6–81.0)	.23	431	58.2 (52.6–63.7)	<.001
≥10 y	405	61.6 (55.4–67.5)	873	80.3 (76.9–83.4)		1,279	70.2 (67.0–73.2)	
Born in US	1,492	63.5 (60.6–66.4)	5,174	80.3 (78.9–81.6)		6,696	78.0 (76.7–79.4)	
Missing/unknown	90	54.5 (42.7–66.0)	173	75.4 (66.9–82.7)		321	69.1 (62.3–75.3)	
County metropolitan status								
Large central metropolitan	695	63.5 (59.2–67.7)	1,708	83.1 (81.0–85.0)	<.001	2,912	73.3 (71.1–75.4)	.002
Large fringe metropolitan	508	63.5 (58.4–68.4)	1,505	81.4 (78.8–83.8)		2,095	79.1 (76.6–81.5)	
Medium/small metropolitan	612	63.1 (57.9–68.0)	2,004	78.0 (75.6–80.2)		2,618	75.0 (72.6–77.3)	
Nonmetropolitan	276	52.7 (46.3–59.0)	1,065	75.5 (71.8–79.0)		1,102	73.9 (70.2–77.4)	
Disability								
Yes	132	51.8 (41.7–61.8)	785	70.1 (66.3–73.7)	<.001	561	61.9 (56.7–66.8)	<.001
No	1,959	62.8 (60.1–65.4)	5,497	81.3 (80.0–82.6)		8,166	76.3 (75.0–77.5)	
Missing/unknown	0	NA	0	NA		0	NA	
Doing errands alone								
At least some difficulty	117	62.0 (51.1–72.1)	618	70.6 (66.2–74.7)	<.001	607	63.0 (58.1–67.7)	<.001
No difficulty	1,974	62.1 (59.5–64.7)	5,662	81.0 (79.7–82.2)		8,120	76.3 (75.0–77.6)	
Missing/unknown	0	NA	—g	—g		0	NA	
Usual source of care^i^								
Yes	1,729	66.8 (64.1–69.4)	5,600	82.7 (81.5–83.9)	<.001	6,973	78.7 (77.3–80.1)	<.001
No	360	41.1 (35.0–47.4)	677	58.1 (53.2–63.0)		1,752	62.7 (59.7–65.6)	
Missing/unknown	—g	—g	—g	—g		—g	—g	
Wellness check within 3 years								
Yes	1,926	66.5 (63.9–69.0)	6,025	82.6 (81.4–83.7)	<.001	8,163	77.9 (76.6–79.1)	<.001
No	154	10.7 (6.2–16.8)	243	19.5 (13.3–27.0)		541	39.6 (34.9–44.6)	
Missing/unknown	—g	—g	—g	—g		—g	—g	
Insurance, aged <65 y								
Private	1,468	67.6 (64.8–70.2)	2,565	83.2 (81.5–84.9)	<.001	5,858	80.0 (78.6–81.4)	<.001
Medicaid/other public	336	58.3 (52.4–64.1)	543	71.4 (66.9–75.6)		1,565	69.8 (66.7–72.7)	
Other coverage	68	69.1 (52.8–82.4)	219	78.5 (71.4–84.4)		331	72.4 (65.9–78.3)	
Uninsured	219	34.5 (27.0–42.6)	229	49.0 (40.4–57.5)		755	55.7 (51.3–60.0)	
Missing/unknown	0	NA	—g	—g		—g	—g	
Insurance, aged ≥65 y^j^								
Private	NA	NA	984	84.9 (82.2–87.4)	<.001	94	85.0 (76.2–91.5)	.11
Medicare + Medicaid	NA	NA	256	72.5 (65.2–78.9)		—g	—g	
Medicare Advantage	NA	NA	1,046	85.1 (82.4–87.5)		—g	—g	
Medicare only	NA	NA	291	70.7 (64.0–76.9)		—g	—g	
Other coverage	NA	NA	120	76.2 (65.4–84.9)		—g	—g	
Uninsured	NA	NA	—g	—g		—g	—g	
Missing/unknown	NA	NA	—g	—g		0	NA	

Abbreviations: AIAN, American Indian or Alaska Native; GED, General Educational Development; NA, not applicable.

a Data source: National Center for Health Statistics, National Health Interview Survey, 2023.

b Numbers are unweighted denominators and percentages are weighted.

c The US Preventive Services Task Force expanded its recommendation for routine breast cancer screening in 2024 to include ages 40–49 years.

d Significance testing was done by using Wald *F* tests and excludes missing/unknown.

e Estimates are age-standardized to the 2000 US standard population by using age groups 50–64 years and 65–74 years for breast cancer screening test use and age groups 21–34, 35–44, and 45–65 years for cervical cancer screening test use.

f AIAN includes AIAN only or in combination.

g Estimate suppressed because it did not meet National Center for Health Statistics reliability standards.

h Significance testing indicates differences between Hispanic and non-Hispanic groups. Information about Hispanic subgroups was available for Mexican/Mexican American respondents and all others combined.

i Respondents reporting that their usual source of care was an urgent care center, drug or grocery store clinic, or emergency department were classified as not having a usual source of care.

j For cervical cancer screening, the older age stratum includes only those aged 65 years because the US Preventive Services Task Force does not recommend routine screening beyond that age.

**Table 2:** Percentage of Screening-Eligible Adults Who Were Up to Date With US Preventive Services Task Force Colorectal Cancer Screening Recommendations, United States, 2023^a^^,^ b

Characteristic	Total			Aged 45–49 y		Aged 50–75 y	
	No.	% (95% CI)	*P* c	No.	% (95% CI)	No.	% (95% CI)
Overall (crude)	14,614	67.4 (66.3–68.4)	NA	1,893	37.1 (34.5–39.7)	12,721	73.4 (72.4–74.4)
Overall (age standardized^d^)	14,614	63.5 (62.5–64.5)	NA	NA	NA	12,721	71.6 (70.5–72.6)
Age, y							
45–49	1,893	37.1 (34.5–39.7)	<.001	1,893	37.1 (34.5–39.7)	NA	NA
50–64	6,966	67.8 (66.5–69.1)		NA	NA	6,966	67.8 (66.5–69.1)
65–75	5,755	82.7 (81.5–84.0)		NA	NA	5,755	82.7 (81.5–84.0)
Sex							
Male	6,774	66.0 (64.6–67.4)	.004	928	37.0 (33.5–40.5)	5,846	72.0 (70.5–73.4)
Female	7,840	68.6 (67.3–69.9)		965	37.1 (33.6–40.8)	6,875	74.7 (73.4–76.0)
Race							
AIAN^e^	254	60.8 (52.2–68.9)	<.001	—f	—f	220	68.8 (59.9–76.7)
Asian	660	58.4 (53.6–63.1)		142	28.5 (20.5–37.7)	518	67.4 (62.0–72.4)
Black/African American	1,720	67.5 (64.8–70.2)		247	41.2 (34.2–48.5)	1,473	72.9 (70.0–75.7)
White	11,261	69.5 (68.4–70.6)		1,300	39.3 (36.2–42.4)	9,961	74.9 (73.8–76.0)
Other single/multiple race	104	63.2 (51.5–73.9)		—f	—f	79	70.7 (57.1–82.0)
Missing/unknown	615	49.6 (44.8–54.5)		145	23.4 (15.0–33.6)	470	58.9 (53.6–64.1)
Ethnicity^g^							
Non-Hispanic	12,857	69.7 (68.6–70.7)	<.001	1,534	39.6 (36.7–42.6)	11,323	75.2 (74.2–76.2)
Hispanic	1,757	53.8 (50.5–57.1)		359	27.0 (21.5–33.1)	1,398	61.9 (58.3–65.3)
Mexican/Mexican American	932	48.3 (43.9–52.7)		201	23.0 (15.9–31.4)	731	56.3 (51.2–61.2)
Other Hispanic	802	60.8 (56.2–65.2)		154	32.6 (23.5–42.7)	648	68.9 (63.9–73.5)
Unknown	—f	—f		—f	—f	—f	—f
Education							
Less than high school	1,299	54.0 (50.4–57.5)	<.001	151	24.8 (16.9–34.1)	1,148	59.7 (55.9–63.4)
High school/GED	3,684	62.4 (60.5–64.2)		436	29.8 (24.7–35.2)	3,248	68.4 (66.5–70.3)
Some college	4,099	69.6 (67.9–71.3)		462	39.6 (34.6–44.7)	3,637	74.9 (73.1–76.6)
College degree	5,469	74.0 (72.7–75.4)		836	43.4 (39.7–47.2)	4,633	81.1 (79.7–82.4)
Missing/unknown	63	51.9 (37.9–65.6)		—f	—f	55	52.3 (37.6–66.7)
% Federal poverty threshold							
≤138	2,353	56.9 (54.2–59.6)	<.001	278	25.4 (19.5–32.1)	2,075	63.2 (60.5–65.9)
>138–250	2,682	63.2 (60.7–65.6)		290	33.4 (26.8–40.4)	2,391	68.6 (66.0–71.1)
>250–400	3,002	65.7 (63.4–67.9)		363	35.9 (29.4–42.8)	2,639	71.2 (68.8–73.6)
>400	6,578	73.0 (71.7–74.3)		962	42.2 (38.6–45.9)	5,616	79.5 (78.1–80.8)
Duration of US residence							
<10 y	185	36.4 (28.1–45.4)	<.001	74	19.2 (10.2–31.4)	111	48.3 (36.5–60.2)
≥10 y	2,097	57.3 (54.7–59.8)		381	27.9 (22.8–33.4)	1,716	65.0 (62.1–67.7)
Born in US	11,898	70.9 (69.8–71.9)		1,362	41.3 (38.2–44.5)	10,536	76.0 (75.0–77.1)
Missing/unknown	434	56.4 (51.0–61.7)		76	34.6 (22.0–49.0)	358	62.4 (56.5–68.0)
County metropolitan status							
Large central metropolitan	4,063	66.6 (64.7–68.5)	.18	621	35.9 (31.5–40.5)	3,442	73.6 (71.6–75.5)
Large fringe metropolitan	3,475	68.8 (66.9–70.7)		469	37.3 (32.6–42.2)	3,006	75.4 (73.5–77.3)
Medium/small metropolitan	4,635	67.6 (65.5–69.5)		557	38.1 (32.9–43.5)	4,078	73.0 (71.1–74.9)
Nonmetropolitan	2,441	65.8 (63.5–68.0)		246	37.0 (29.8–44.6)	2,195	70.3 (67.9–72.7)
Disability							
Yes	1,612	70.6 (67.9–73.2)	.01	106	47.6 (36.2–59.2)	1,506	72.7 (70.0–75.3)
No	13,002	67.0 (65.9–68.0)		1,787	36.5 (33.9–39.2)	11,215	73.5 (72.4–74.5)
Missing/unknown	0	NA		0	NA	0	NA
Doing errands alone							
At least some difficulty	1,230	69.2 (66.1–72.1)	.22	95	50.3 (37.6–63.0)	1,135	71.3 (68.1–74.4)
No difficulty	13,381	67.2 (66.1–68.3)		1,798	36.4 (33.8–39.1)	11,583	73.6 (72.5–74.6)
Missing/unknown	—f	—f		0	NA	—f	—f
Usual source of care^h^							
Yes	12,705	71.5 (70.5–72.5)	<.001	1,508	40.9 (38.0–43.8)	11,197	77.0 (76.0–78.0)
No	1,898	41.1 (38.4–43.9)		384	22.3 (17.7–27.4)	1,514	47.4 (44.3–50.6)
Missing/unknown	—f	—f		—f	—f	—f	—f
Wellness check within 3 yrs							
Yes	13,731	70.6 (69.6–71.6)	<.001	1,700	39.7 (37.0–42.5)	12,031	76.4 (75.4–77.4)
No	841	18.0 (15.0–21.5)		183	12.3 (7.2–19.1)	658	20.2 (16.6–24.2)
Missing/unknown	—f	—f		—f	—f	—f	—f
Insurance, aged <65 y							
Private	6,398	64.7 (63.3–66.0)	<.001	1,395	40.1 (37.1–43.1)	5,003	72.5 (71.0–73.9)
Medicaid/other public	1,149	55.6 (52.2–59.0)		236	38.0 (30.4–46.1)	913	61.3 (57.6–65.0)
Other coverage	577	69.9 (65.3–74.2)		69	43.0 (29.8–57.1)	508	74.2 (69.4–78.7)
Uninsured	716	23.8 (19.9–28.1)		188	14.3 (8.0–22.8)	528	28.1 (23.3–33.2)
Missing/unknown	—f	—f		—f	—f	—f	—f
Insurance, aged ≥65 y							
Private	2,063	84.4 (82.4–86.3)	<.001	NA	NA	2,063	84.4 (82.4–86.3)
Medicare + Medicaid	469	76.4 (71.4–80.9)		NA	NA	469	76.4 (71.4–80.9)
Medicare Advantage	2,114	85.6 (83.7–87.4)		NA	NA	2,114	85.6 (83.7–87.4)
Medicare only	633	75.4 (71.3–79.2)		NA	NA	633	75.4 (71.3–79.2)
Other coverage	421	83.1 (78.1–87.4)		NA	NA	421	83.1 (78.1–87.4)
Uninsured	—f	—f		NA	NA	—f	—f
Missing/unknown	—f	—f		NA	NA	—f	—f

Abbreviations: AIAN, American Indian or Alaska Native; GED, General Educational Development; NA, not applicable.

a Data source: National Center for Health Statistics, National Health Interview Survey, 2023.

b Numbers are unweighted denominators and percentages are weighted.

c Significance testing was done by using Wald *F* tests and excludes missing/unknown.

d Estimates are age-standardized to the 2000 US standard population by using age groups 45–54, 55–64, and 65–75 years for the estimate for all ages and age groups 50–54, 55–64, and 65–75 years for the estimate for those aged 50–75 years.

e AIAN includes AIAN only or in combination.

f Estimate suppressed because it did not meet National Center for Health Statistics reliability standards.

g Significance testing indicates differences between Hispanic and non-Hispanic groups. Information about Hispanic subgroups was available for Mexican/Mexican American respondents and all others combined.

h Respondents reporting that their usual source of care was an urgent care center, drug or grocery store clinic, or emergency department were classified as not having a usual source of care.

For women aged 50 to 74 years, breast cancer screening test use varied significantly with all sociodemographic and health care access factors, except Hispanic ethnicity and duration of US residence. Patterns among women in their forties were generally similar to those among older women, although use was lower in the younger (40–49 y) age group. Cervical cancer screening test use varied significantly for all factors except health insurance coverage among women aged 65 years, while CRC screening test use varied by all factors except county metropolitan status and difficulty doing errands. For those of screening age, in addition to CRC screening test use among adults aged 45 to 49 years (37.1%), estimates were less than 50% for those without wellness checks within 3 years (18.0%–39.6% for all screening types) or usual sources of care (41.1% for CRC), uninsured people younger than 65 years (49.0% for breast and 23.8% for CRC), those reporting Mexican/Mexican American ancestry (48.3% for CRC), and those residing in the US fewer than 10 years (36.4% for CRC). For breast, cervical, and CRC screening test use, those lacking transportation or experiencing food insecurity, difficulty paying housing/utility bills, or medical financial hardship were generally less likely to be up to date (Table 3).

**Table 3:** Percentage of Screening-Eligible Adults Up to Date With US Preventive Services Task Force-Recommended Breast, Cervical, and Colorectal Cancer Screening Test Use, by Transportation, Food Insecurity and Cost Barriers^a^^,^ b

Characteristic	Breast cancer screening, aged 50–74 y			Cervical cancer screening, aged 21–65 y			Colorectal cancer screening, aged 45–75 y		
	No.	% (95% CI)	*P* c	No.	% (95% CI)	*P* c	No.	% (95% CI)	*P* c
Lack of reliable transportation^d^									
Yes	434	67.0 (61.6–72.0)	<.001	668	68.7 (64.0–73.2)	.002	919	59.5 (55.4–63.6)	<.001
No	5,674	81.0 (79.7–82.2)		7,727	76.2 (74.9–77.5)		13,253	68.1 (67.1–69.2)	
Missing/unknown	174	75.7 (66.5–83.5)		332	69.4 (63.1–75.1)		442	60.1 (54.9–65.2)	
Food security									
High security	5,179	81.2 (79.9–82.4)	<.001	6,895	77.0 (75.5–78.4)	<.001	12,258	68.8 (67.7–69.8)	<.001
Marginal security	358	77.9 (72.0–83.0)		618	71.3 (66.7–75.5)		741	63.2 (58.7–67.6)	
Low security	316	76.7 (70.8–81.9)		502	68.9 (63.8–73.7)		635	58.0 (53.2–62.7)	
Very low security	258	63.1 (55.8–69.9)		396	67.3 (61.4–72.9)		541	57.4 (51.9–62.7)	
Missing/unknown	171	77.0 (68.5–84.1)		316	69.5 (63.0–75.5)		439	60.8 (55.5–65.8)	
Unable to pay mortgage/rent/utility bills in past 12 months									
Yes	421	66.6 (61.0–71.9)	<.001	857	71.3 (67.4–75.0)	.02	903	56.3 (52.4–60.2)	<.001
No	5,676	81.2 (79.9–82.4)		7,530	76.2 (74.8–77.5)		13,249	68.4 (67.4–69.5)	
Missing/unknown	185	73.8 (64.7–81.6)		340	67.8 (61.7–73.5)		462	59.5 (54.4–64.5)	
Problems paying medical bills in past 12 months									
Yes	705	73.0 (68.9–76.8)	<.001	1,037	73.8 (70.3–77.0)	.28	1,479	61.7 (58.6–64.7)	<.001
No	5,568	80.9 (79.6–82.1)		7,673	75.6 (74.3–77.0)		13,110	68.1 (67.0–69.1)	
Missing/unknown	—e	—e		—e	—e		—e	—e	
Worry about paying medical bills if got sick/had an accident									
Very worried	911	71.8 (68.1–75.2)	<.001	1,473	70.0 (66.9–72.9)	<.001	1,849	55.3 (52.2–58.3)	<.001
Somewhat worried	1,904	79.1 (76.9–81.2)		3,035	77.0 (74.9–79.0)		4,261	65.5 (63.7–67.2)	
Not worried	3,461	82.8 (81.3–84.3)		4,206	76.2 (74.5–77.9)		8,478	71.4 (70.2–72.5)	
Missing/unknown	—e	—e		—e	—e		—e	—e	
Delayed/did not get medical care because of cost in past 12 months									
Yes	512	65.5 (60.4–70.4)	<.001	960	68.5 (64.9–71.9)	<.001	1,063	53.0 (49.2–56.8)	<.001
No	5,768	81.3 (80.1–82.6)		7,761	76.3 (74.9–77.6)		13,544	68.6 (67.6–69.5)	
Missing/unknown	—e	—e		—e	—e		—e	—e	

a Data source: National Center for Health Statistics, National Health Interview Survey, 2023.

b Numbers are unweighted denominators and percentages are weighted.

c Significance testing was done by using Wald *F* tests and excludes missing/unknown.

d Includes lack of reliable transportation that kept respondents from medical appointments, meetings, work, or getting things in the past 12 months.

e Estimate suppressed because it did not meet National Center for Health Statistics reliability standards.

Age-standardized estimates of mammography use were similar in 2019 and 2021 (76.2% [95% CI, 74.9%–77.5%] and 75.6% [95% CI, 74.4%–76.8%], respectively) but somewhat higher in 2023 (79.8% [95% CI, 78.5%–81.1%]; *P* < .001) (Figure). Cervical screening test use was lower in 2023 (75.8% [95% CI, 74.6%–77.0%]) than in 2021 or 2019 (79.1% [95% CI, 77.9%–80.3%] in 2021 and 80.0% [95% CI, 78.9%–81.1%] in 2019; *P* < .001). In 2023, the age-standardized percentage of those up to date with CRC screening test use was 63.5% (95% CI, 62.5%–64.5%) among adults aged 45 to 75 years. In 2021, the estimate for adults aged 50 to 75 years was 70.3% (95% CI, 69.3%–71.4%). Estimates for those aged 50 to 75 years were similar between years (70.3% [95% CI, 69.3%–71.4%] for 2021 and 71.6% [95% CI, 70.5%–72.6%] for 2023). Colonoscopy use within 10 years was also similar over time for those aged 50 to 75 years (61.0% [95% CI, 60.0%–62.1%] for 2019, 61.8% [95% CI, 60.7%–62.9%] for 2021, and 61.3% [95% CI, 60.2%–62.4%] for 2023); the 2023 colonoscopy estimate including ages 45 to 75 years was 53.4% (95% CI, 52.3%–54.4%) (not shown). FOBT/FIT use was similar in 2021 and 2023 (10.0% [95% CI, 9.3%–10.8%] in 2021 vs 9.3 [95% CI, 8.7%–9.9%] in 2023). FIT/DNA use was 10.0% (95% CI, 9.4%–10.6%) in 2023 and 8.3% (95% CI, 7.7%–8.9%) in 2021 (not shown).

**Figure:**
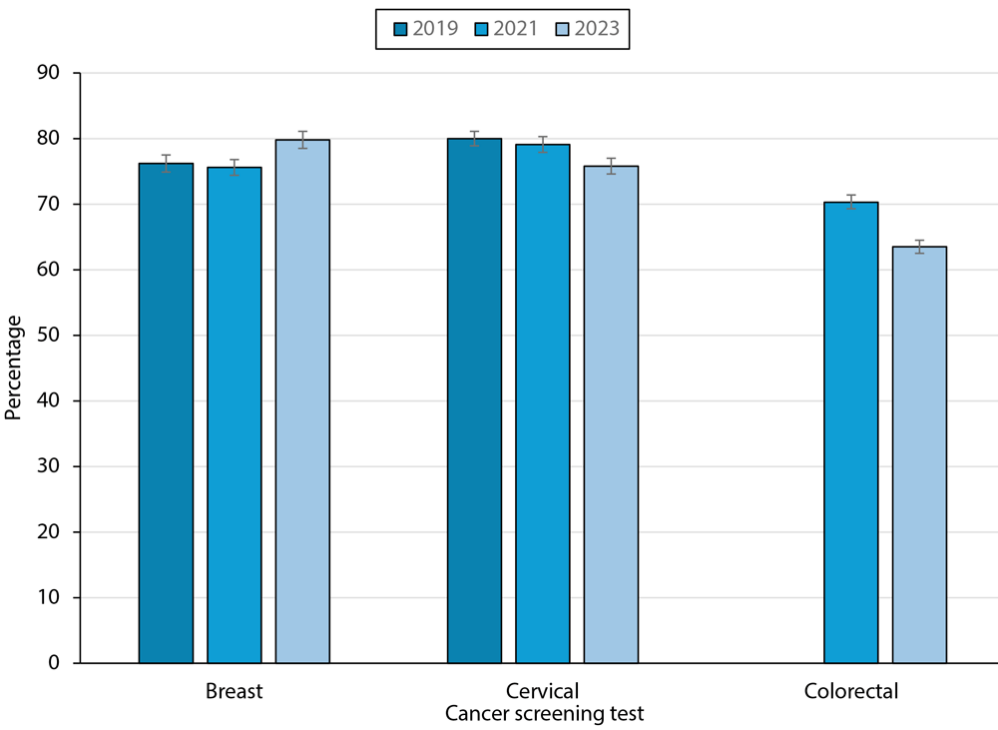
Age-standardized (to the 2000 US standard population) estimates of being up to date with breast, cervical, and colorectal screening test use based on US Preventive Services Task Force recommendations, by year. In 2023, percentages up-to-date increased for breast screening test use and decreased for cervical and colorectal screening test use. Percentages are weighted. Colorectal cancer screening estimates are based on US Preventive Services Task Force-recommended screening ages for each year (ages 50–75 years for 2021 and ages 45–75 years for 2023). The colorectal screening estimate for 2019 is not shown because of differences in colorectal screening test use data available in that year. Data source: National Health Interview Survey.

**Table:** 

Screening test	% (95% CI)		
	2019	2021	2023
Breast cancer	76.2 (74.9–77.5)	75.6 (74.4–76.8)	79.8 (78.5–81.1)
Cervical cancer	80.0 (78.9–81.1)	79.1 (77.9–80.3)	75.8 (74.6–77.0)
Colorectal cancer	Not applicable	70.3 (69.3–71.4)	63.5 (62.5–64.5)

## Discussion

In 2023, most screening-eligible adults were up to date with breast, cervical, and CRC screening test use, with estimates ranging from 67% to 80%. Mammography use approximated the HP2030 target of 80.3%. Most women aged 40 to 49 years had also received a mammogram within 2 years, although their use was lower than for older women (62.1% vs 80.0%). The estimate for women in their forties reflects use before USPSTF recommended expanding routine screening to this age group in 2024 (2) and therefore may reflect individualized preferences or physician recommendations to start screening before age 50 years. An analysis of 2022 Behavioral Risk Factor Surveillance System (BRFSS) data reported slightly lower estimates of mammography use (59.1% for those aged 40–49 y and 76.5% for those aged 50–74 y) (21). For CRC screening test use, the 2023 estimate, which is based on the age range expanded to include those aged 45 to 49 years, who had lower screening estimates (37.1%), was lower than the 2021 estimate and was also below the HP2030 target of 68.3%. Caution interpreting cervical cancer screening test estimates may be warranted given possible misclassification when self-reporting screening (22,23). For example, underreporting might be possible because women may not know if HPV tests were performed during screening (23). In 2023, NHIS asked broadly about cervical cancer screening and not about tests received, although the survey question did state that Pap and HPV tests were the 2 cervical screening tests. This approach might reduce underreporting but could make overreporting more likely. Previously reported age-standardized estimates for 2019 and 2021 based on questions about type of test received were somewhat lower (76.8% [95% CI, 75.6%–77.9%] and 75.5% [95% CI, 74.2%–76.7%], respectively) (4,11).

Although most adults of screening age were up to date, 20% to 33%, or 1 in 5 adults to 1 in 3 adults, were not. Across screening types, health care access was strongly associated with being up to date, as in previous studies (4,13,21,24,25). Groups with less access generally had lower screening test use, often with more than half of respondents in these groups not up to date. Low estimates were found among those without recent wellness visits. Others have reported that wellness visits potentially have not returned to prepandemic levels (26). The Centers for Disease Control and Prevention’s (CDC’s) National Breast and Cervical Cancer Early Detection Program helps increase access by providing free or low-cost breast and cervical cancer screenings to qualifying women (www.cdc.gov/breast-cervical-cancer-screening). For CRC screening, in addition to health care access, low use was also found among several other groups. CDC’s Colorectal Cancer Control Program works to implement evidence-based interventions to increase screening, focusing on clinics that serve people with lower incomes and where fewer than 60% of patients are up to date with screening (www.cdc.gov/colorectal-cancer-control/about/how-crccp-increases-screening.html).

Lacking reliable transportation, food insecurity, difficulty paying housing/utility costs, and medical financial hardship were factors that consistently had lower screening test use. Although findings in this descriptive study were not adjusted for possible confounders, they are consistent with previous evidence (21,27,28). In an analysis of 39 jurisdictions in the 2022 BRFSS examining similar barriers and measures of social and emotional support, isolation, satisfaction and stress, mammography use decreased as the number of such barriers increased, from 83.2% among those with no barriers to 65.7% among those with more than 3 barriers (21). Similar to our findings, lacking reliable transportation, receiving supplemental nutrition assistance, and cost barriers to care were associated with lower mammography use (21). Others have reported that among women receiving mammograms, barriers to receiving health care were associated with lower likelihood of receiving mammograms on schedule, with being uninsured and health care cost the barriers most frequently reported (27). Similar findings have been reported for CRC screening (28), although the association may vary by type of screening test (29). Beyond screening, such barriers could have implications for follow-up diagnostic care and treatment (27) and have been associated with increased mortality in cancer survivors (17). Continued monitoring can help determine whether the prevalence of these barriers is changing over time, for which groups, and potential effects on screening, other health care services, and health outcomes.

Our findings suggest little change in estimates of breast and cervical screening test use from 2019 to 2021, consistent with previous studies (4,8). Earlier evidence about mammography suggests little change from 2005 to 2018 (8,12) or 2012 to 2020 (25). The increase in mammography in 2023 is notable given previous evidence of stable mammography use for years. Cervical cancer screening test use decreased somewhat in 2023. Studies preceding the 2019 NHIS survey redesign reported declines in cervical screening use from the early 2000s to 2013–2015 (12,24), with some reporting an increase through 2016–2018 (8,12); our analysis suggests similar use in 2019 and 2021. Some have suggested that declines in Pap test use alone before 2019 might be attributed to factors such as lengthening screening intervals over time (30,31) and possibly HPV vaccination, with mixed evidence regarding its influence on screening use (30). Others have reported that HPV vaccination is an unlikely reason for not being up to date with cervical cancer screening and that among women aged 30 to 65 years, lack of access decreased as a self-reported reason for not being up to date from 2005 to 2019, while lack of knowledge and no provider recommendation as self-reported reasons increased (32). Age-standardized estimates of CRC screening test use were 63.5% (95% CI, 62.5%–64.5%) in 2023 with the introduction of the lower age threshold of 45 years, compared with 70.3% (95% CI, 69.3%–71.4%) among adults aged 50 to 75 years in 2021. Before the COVID-19 pandemic, CRC screening test use had been increasing (8,12); we found no increase in 2023 even for those aged 50 to 75 years. Findings were largely driven by colonoscopy use, which was similar over time for those aged 50 to 75 years (61.0%–61.8%) and 53.4% for those aged 45 to 75 years in 2023. FOBT/FIT use was similar in 2021 and 2023, suggesting that previously reported increases during the COVID-19 pandemic (4,8) may have been sustained; FIT-DNA use may have increased modestly. Differences between these findings and studies reporting declines in screening during the COVID-19 pandemic (5,26) might be explained at least in part by differing time frames; we examined use within recommended intervals while others examined use within 1 year (5,8,26). Some studies reported that prior year use was lower in 2020–2021 than in 2018–2019, although as in our study percentages up to date with these screenings were similar (8,26), perhaps due to reductions in screening overuse (8). For all screening types, refinements in survey methods might contribute to differences over time (16); the extent to which refinements may have contributed to differences is unknown and limits conclusions based on these comparisons.

### Strengths and limitations

Study strengths include a large, nationally representative data set that includes people with and without health care access. Limitations include self-reported data subject to recall bias. Response rates in 2021 and 2023 were lower than in 2019 (50.9% and 47.0% vs 59.1%, respectively); however, weights are adjusted for nonresponse (16). Findings are unadjusted for confounding, which was out of scope for this descriptive report. Detail about type of cervical screening tests received was unavailable in 2023, precluding comparison with the HP2030 target. Analysis of screening test data from future survey years could help monitor progress toward the target. Based on an analysis of annual test use in claims data for commercially insured women aged 30 to 64 years, Qin et al reported that co-testing increased and Pap test alone decreased from 2013 to 2019 (30). In 2019, co-testing was the most common cervical cancer screening test option, and HPV testing alone was infrequent. An analysis of screening data from a state registry reported similar findings for co-testing and HPV testing alone between 2008 and 2019 (33). Differences in CRC screening questions in 2019 limited comparisons over the 3-year period. Diagnostic tests could have been included in the analysis, consistent with previous studies and Healthy People targets (3,4,11,13,34,35). Finally, the threshold for the NHIS disability status indicator (based on the Washington Group’s Short Set on Functioning [16]) may not capture milder conditions.

### Conclusion

In 2023, most adults were up to date with breast, cervical, and CRC screening test use; however, 1 in 3 adults to 1 in 5 adults were not. Differences in screening test use existed, including for those with less health care access and other barriers. Future monitoring can help determine if changes in screening test use continue and track progress toward national targets.
